# What is Popular Gets More Popular? Exploring Over-Time Dynamics in Article Readership Using Real-World Log Data

**DOI:** 10.1080/1461670X.2024.2411334

**Published:** 2024-10-14

**Authors:** Damian Trilling, Roeland Dubèl, Rupert Kiddle, Anne C. Kroon, Zilin Lin, Mónika Simon, Susan Vermeer, Kasper Welbers, Mark Boukes

**Affiliations:** a Department of Communication Science, University of Amsterdam, Amsterdam, Netherlands; b Language, Literature and Communication, Vrije Universiteit Amsterdam, Amsterdam, Netherlands; c Strategic Communication, Wageningen University, Wageningen, Netherlands; d Department of Communication Science, Vrije Universiteit Amsterdam, Amsterdam, Netherlands

**Keywords:** Online news use, feedback loops, log data, news consumption, regional news, audience metrics

## Abstract

Online news can be shared and promoted via social media, mobile push messages, newsletters, “most read” boxes, or the like. This can result in feedback loops, in which views attract even more views. Using full click logs for five regional newspapers in the Netherlands, spanning $N_{\mathrm{views}}=$12,108,263 views of $N_{\mathrm{articles}}=$ 17,982 articles for each minute over the course of 13 weeks, we shed light on potential feedback loops. While article placement and promotion decisions indeed increase their views, we find these effects to be short-lived, contradicting the feedback-loop hypothesis. Exceptions in line with the feedback-loop hypothesis mostly concern social media: If an article is spread via social media, it is not only clicked more, but also clicked on for a longer period of time.

In contemporary online news environments, users typically consume news by the item rather than per “issue”. Hence, scholars and practitioners want to understand which articles readers choose to engage with over others. These observations are fundamental to understanding selective exposure and selective avoidance (e.g., Zuiderveen Borgesius et al. 2016), and how audience metrics influence journalistic decision making (e.g., Lamot 2021). To understand which articles attract the most views (clicks),^1^ scholars have considered topics (e.g., Tewksbury 2003), linguistic features (e.g., Kuiken et al. 2017), and related concepts. Next to such *static* features, we investigate the *dynamics* of an article's popularity over time.

Popularity can be understood as a form of real-time audience feedback. When defined as the number of views an article receives, the popularity of an article can drive further engagement. Many newsrooms use “popularity-based filtering” to highlight frequently viewed articles (e.g., Møller 2022). Thus, views at any point in time $t_{i}$ can positively influence views at $t_{i+1}$. Alternatively, popularity can operate as an engagement cue for audiences. Readers often regard a “Most Read” label as a cue for an article's importance (Messing and Westwood 2014). Moreover, newsrooms may influence the popularity of articles by performing actions to promote specific articles, which may further sustain these dynamic effects.

We aim to disentangle the reciprocal interplay (i.e., the feedback loops) between the way content is communicated to the user (e.g., via website placement, editorial recommendations, push notifications, or external social media promotion) and article popularity (views). This is important as the provision of real-time user feedback on news sites interferes with the traditional agenda setting power of journalists. By informing others what is important and worth reading, audiences themselves become agenda-setters (E. J. Lee and Tandoc 2017). Previous empirical work indicates that online news consumption patterns largely follow supply patterns (Makhortykh et al. 2021). Amplified by popularity cues and popularity-based recommendation, the dynamic transformation of the supply could lead to news diets that lack the breadth and variety necessary for an informed citizenry. If an initial audience preference for less relevant (from a democratic point of view) content leads to a reinforcing process which restricts the visibility of content that is more relevant to public affairs, then this may be detrimental to the capacity of journalism to sustain an informed citizenry conducent to healthy democracy.

To evaluate potential feedback loops in news article popularity, we ask: How substantial is the self-reinforcing effect of popularity? Do articles that start popular, gain a compounding advantage over those that do not? Can articles that gain little traction after being published make up for this later? We also aim to understand how the role of journalistic decisions—for instance, the decision to publish an article in multiple selections, or to editorially recommend it—contributes to the dynamics of popularity.

We analyze log data of five regional newspapers in the Netherlands covering a period of 13 consecutive weeks. Mediahuis, the publishing company owning the newspapers, provided us with *all* visits to *all* articles for *every* minute, allowing us to reconstruct the trajectory of visits. While communication research mainly focuses on national newspapers, regional newspapers play an important role in citizens' news diets; in the Netherlands such newspapers circulate at levels only a third to a fifth lower than national newspapers (Bakker and Scholten 2019). Our dataset is also unique in that the exact same articles are often published across different newspapers within it; allowing for more precise estimation of popularity dynamics within this historically understudied source of news.

## Theoretical Background and Related Research

Early research on clicks within news sites demonstrated that readers do not always click on what editors find important (Tewksbury 2003). Traditionally, it is assumed that the newspaper agenda largely sets the audience's agenda, but this idea increasingly is under pressure. Hence, Shehata and Strömbäck (2013) summarize concerns that increasing user choices may weaken the media's agenda-setting power. There have always been feedback loops between journalists and audiences, such that journalists also adopt the agenda based on perceived audience preferences (Trilling 2024). But *real-time* audience feedback intensifies this reciprocal process, shifting more agenda-setting power to the audience (E. J. Lee and Tandoc 2017), especially if the feedback provided by clicks *automatically* influences the positioning of articles. In several countries, newsrooms already use ranking logics on their homepage, largely driven by popularity metrics (Borchgrevink-Brækhus 2022).

To better understand the popularity dynamics of online news, we first review the literature to identify which factors drive click volume.

### Recency and Currentness

Despite disagreement on the exact boundaries of what exactly can be defined as “news” (see e.g., Costera Meijer 2020; Wall 2015), one definitional element is largely uncontested: news regards unfolding current affairs, or in other words, news has to be new.

But what qualifies as *new*? From a strictly user-centric perspective, anything unknown to a given user is “new”. Yet, this would be a too broad of a definition, as it would imply that news never becomes outdated. In a traditional media environment, one may resort to the concept of the issue: today's paper is new, yesterday's paper is old. But in today's unbundled media environment, this heuristic is unavailable. There are no fixed publication times, and although an article eventually disappears from the top of the page, it typically stays available—albeit less visible—for an extended period.

Yet, a rule of thumb that news ceases to be new after roughly 24 hours is still often used. Developing a popularity prediction system for the *Washington Post*, Keneshloo et al. (2016) predicted “…the number of page views that a news article [would] receive within the first day since its publication” ( 441–442), signaling that also online, the newspaper still assumes that its readers are interested in todays, and not yesterday's, news. Supporting this, Castillo et al. (2014, 219) estimate the shelf-life—which they define as the duration at which 90% of all visits that an article will ever receive have occurred—for news articles at Al Jazeera as 1 day and 16 hours, and for in-depth articles as 2 days and 16 hours. Moreover, they find that the half-life (i.e., the median) of an article is reached at 8 hours for news and 20 hours for more in-depth coverage. Bright and Nicholls (2014) find slightly shorter news cycles in major British news sites, estimating that—depending on the outlet—articles are a removed on from the front page on average between 10 and 20 hours after publication.

Nevertheless, quasi-random visits can still occur even years after publication and attempts to leverage the “long tail” through links or automated recommendations can drive delayed views. This increases the time a user spends on a news site at little to no cost, and at content may be relevant again, for instance at anniversaries. We ask:

RQ1 How do article views develop over time after publication of the article?

### Short vs. Long Form

As discussed in the previous section, (short-form) news items may have a shorter shelf-life than in-depth articles (Castillo et al. 2014). Journalism scholars tend to distinguish between these genres, often explicitly mentioning their length as important distinction (Donkers, Markhorst, and Smits 2010). The latter tend to abstract from a specific news event—for instance, Trilling and van Hoof (2020) address how an analytical background piece can cover several events, potentially dispersed in time. As such, long form journalism may offer value to readers well after its publication date. Hence, the length of an article should be a reasonable proxy for the form of journalism and thus how long an article retains some level of popularity. We expect:

H1 There is a positive relationship between the length of an article and its shelf-life.

### Placement and Promotion

When selecting what to read, users are aided by what Donsbach (1991) calls “formal emphasis”. A more prominent placement, he shows, is so powerful that it can even override readers' general preference for likeminded content. While his work focused on print newspapers, in particular the positioning of an article and the size of the headline ( 171), we assume that emphasis devices on news website have similar effects. We consider where on a website links to an article are placed, and the use of specific categories and labels.

Indeed, links on the front page gains most attention from online news audiences (von Krogh and Andersson 2016), but an article can be present on one or more pages (typically for different thematic sections),^2^ which increases the likelihood that audiences encounter it. Additionally, news sites often have sections or features in which they present articles that are marked as highlighted, recommended, or selected by the editors—a very explicit form of Donsbach's “formal emphasis”. Such designations typically also imply that these articles are presented more prominently, and often also are accessible via more routes—for instance, both via their original placement on the website and via a section or feature that gathers such recommendations. We hypothesize:

H2 There is a positive relationship between the number of sections in which an article is published and its number of views.
H3 There is a positive relationship between being featured (a) on the home page, (b) as specifically highlighted, or (c) as recommended by the newsroom, and the number of views of an article.

We do not have specific expectations regarding the effect of different sections on article popularity:

RQ2 What is the effect of section placement on (a) the number of views and (b) the shelf-life of an article?

### Is Popularity Self-Reinforcing?

**Figure 1.**
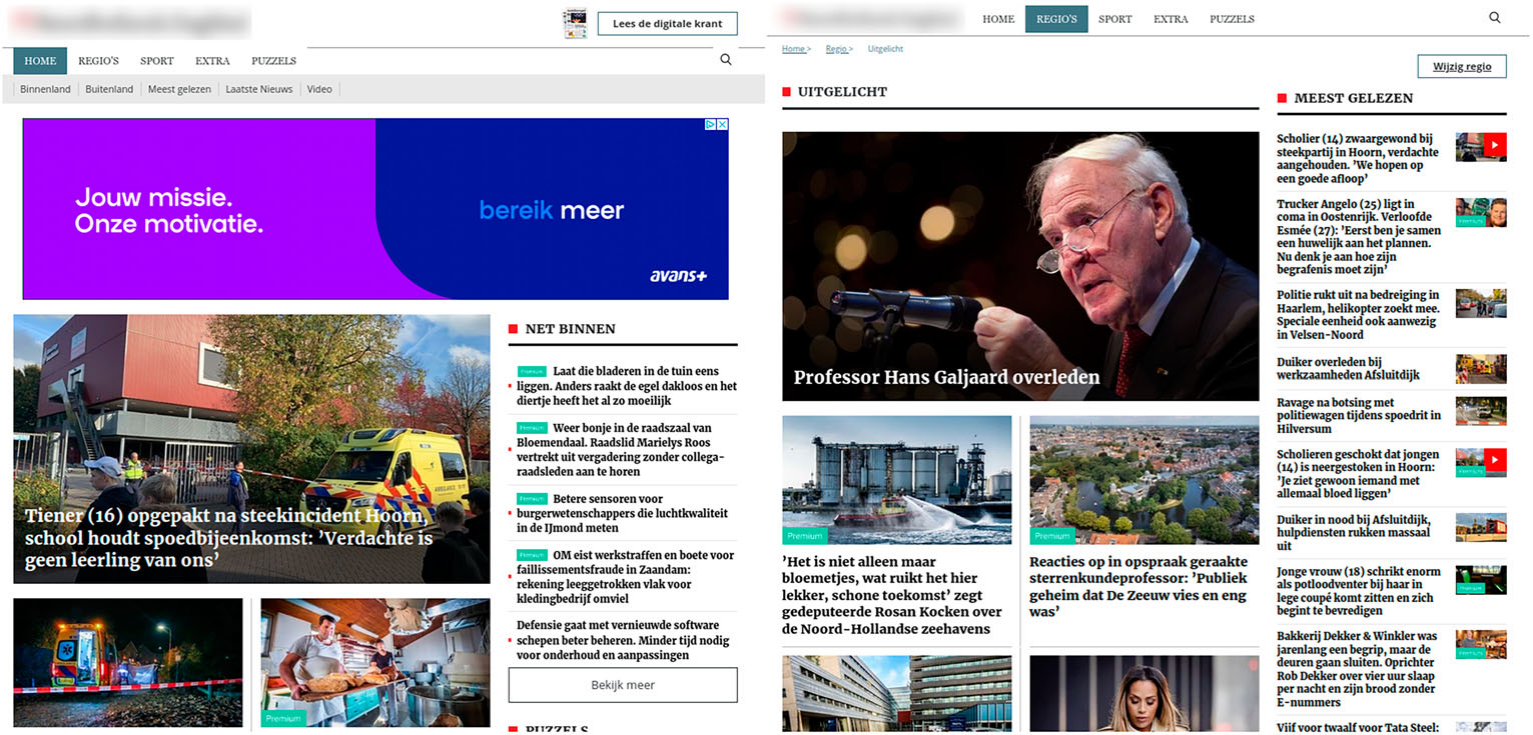
Multiple ways of accessing an article: via the homepage (left), or via specific pages and sections like “In the spotlight” (“uitgelicht”) or “Most read” (“Meest gelezen”, both right). Other pages, which can be selected via the menu bar on top, include specific pages per town, or traditional journalistic sections like “foreign news”, “domestic news”, “sports”, etc.

Popularity bias is not unequivocally seen as negative. For instance, publishers often fuel this process by adding “most read” boxes to their front pages (Figure 1). Without other information about a visitor, it is a reasonable assumption that what many others have read, will also be interesting to the new readers. Visitors indeed read “most read” labels as a cue for the article's importance (Messing and Westwood 2014), and some welcome the implied shift of some agenda-setting power from editors to audiences.

From another perspective, though, popularity bias is problematic. When news websites prioritize popular news articles, some articles coincidentally get a head-start (e.g., due to randomness or slightly better timing) and thereby may enjoy a re-amplifying advantage. This could shape public perceptions as other important issues that may be less popular but equally deserving of attention are potentially overshadowed. These articles are being crowded out by feedback-loop effects that are driven partly by timing and chance, rather than by an equitable assessment of relevance and newsworthiness.

It is far from clear what article popularity dynamics look like in a non-experimental, large-scale, real-world setting. For instance, Loecherbach et al. (2021) show that users often do not comply with article recommendations, in particular when *saturation* occurs: They simply have already read enough about a certain topic, or maybe even have already read the same article. Thus, it is not clear yet to what extent readers engage with “most read” labels, or with articles that are in other ways advantaged by their popularity. To address this deficiency, we explore:

RQ3 To what extent does article popularity in the first hours after publication shape the overall viewing trajectory?

### Boosting Views

Newsrooms also influence popularity dynamics through external promotion. While direct visits are the main driver of news site visits, a substantial share of traffic comes from search and social media (e.g., Vermeer et al. 2020; Wojcieszak et al. 2022). Exact values vary considerably between different media systems and outlets, but it is reasonable to expect that articles that receive greater social media engagement may also accumulate more views on their original news websites. Similarly, articles that are featured in the outlet's email-newsletter (a hardly ever studied yet important element in news dissemination; Kuiken et al. 2017) can expect more clicks. Another understudied news dissemination channel is mobile push notification, “a remarkably consistent part of news distribution channels among the European outlets” (Wheatley and Ferrer-Conill 2021, 710).

Boosting views through these mechanisms, can create a feedback loop wherein increased audience engagement with certain news content leads to further coverage and emphasis on these topics by the media. This reinforcement could strengthen agenda-setting effects—as the continued emphasis on certain topics on, for instance, social media platforms, can perpetuate their significance in the public agenda.

While is it trivial to argue that promotion via social media would lead to more views of an article, whether this exercise results in a short-lived spike or sustained (reinforcing) effect is of great practical and theoretical interest. Therefore, we ask:

RQ4 How does external traffic from (i) social media, (i) newsletters, and (iii) mobile push notifications influence the viewing trajectory of an article?

## Data and Methods

Mediahuis shared their real-world view-logs from weeks 15–27 in 2021 (April–June) for five regional newspapers with us ($N_{\mathrm{views}}=$13,369,338 views of $N_{\mathrm{articles}}=$ 19,685 articles). All newspapers use the same content management system, and each article has a unique ID across all five websites. Hence, we were able to identify identical articles published by multiple outlets.

### Viewing Logs

The log files contained the number of views of an article in any given minute, split up by referrer, distinguishing between views originating from taps on mobile push messages, newsletter links, social media, etc. Due to their sparsity (almost no article receives views every single minute), we aggregated our data by the hour. Before aggregation, we stripped URL parameters to avoid identical articles being counted as different.

### Article Metadata

#### Timestamps

To obtain the time elapsed since publication, we subtracted the publication date and time from the timestamps in the log files.

#### Section trees

Each article comes with a list of (sub-) sections. One article can be published at multiple places, including the homepage and/or sub-pages. These can be pages for specific sections (such as sport) or specific regions (e.g., a municipality), at one or more of the outlets. We inferred in which section(s) an article was placed, as well as the number of sections. Sections are not necessarily presented at comparable places: The homepage counts as a section, the regional pages that can be accessed through a menu count as sections, but also dynamic elements like “Chosen by the editors” or “Highlighted” boxes constitute sections.

#### Article length

We measured character count, as in-depth background stories tend to be longer than short news items.

#### Control variables

In our regression models, we control for several factors. These include:

- a dummy variable for each newspaper;
- a dummy variable for each day of the week;
- a dummy variable for each the time of the day;^3^
- the author: we recoded the bylines into four categories: journalists identified by name, “from our reporters”, “from our newsroom”, or a press agency. These can be seen as a proxy for the exclusivity of the article;
- a dummy variable indicating whether the article was behind a paywall;
- a dummy variable indicating whether the article featured additional inline media, such as embedded videos.

### Analytical Approach

We consider two outcome metrics: the absolute number of views and the shelf-life. We follow Castillo et al. (2014) and define shelf-life as the elapsed duration at which 90% of all visits that an article will ever receive (in the dataset) has occurred. This measure is insensitive to outlier visits and gives a reasonable approximation of the point when an article is not considered relevant by audiences any more.

We estimate regression models to check whether our findings hold once other variables are controlled for. In particular, since we can identify when an article was published across multiple newspapers, we make use of multilevel modeling (MLM) and consider publishers to be nested within identical articles. A random intercept model can then be applied using the following equation:

(1) $$y_{\mathrm{ij}}=\gamma_{00}+u_{0j}+{\text{β}}_{1j}x_{1\mathrm{ij}}+\cdots +\epsilon_{\mathrm{ij}}$$

We deconstruct the overall intercept (${\text{β}}_{0j}$) of our popularity measures (total views and shelf-life) into a fixed part ($\gamma_{00}$), i.e., a part that is constant across all cases, and a random part ($u_{0j}$), i.e., a part that varies across article clusters. By doing so we take into account that some articles receive more engagement (i.e., have a higher “popularity” intercept), which allows us to better isolate what the effect is of the features of interest (being published on the front page, the length of the article, etc.).

This approach also means that we can only include articles that have been published by more than one newspaper, reducing the number of observations in these models to $N_{\mathrm{MLM}-\mathrm{models}}=$37,028, compared to $N_{\mathrm{total}}=$ 46,550 which we use in the other tests. In the Supplementary Materials we present alternative model specifications that disregard the nested structure and include all observations.

A negligible portion of our data (0.62%, *n* = 289) of all publications have been tracked for less than 24 hours, as they were published at the very end of our data collection period.

All data wrangling and analysis was done in Python. While we cannot share the publisher's proprietary data, we share our analysis scripts here: https://github.com/Roeland-Dubel/news_popularity.

## Results

### General Overview

We analyze N=19,685 unique articles, constituting N=46,550 published articles, due to the practice of re-using content across multiple news sites. Nevertheless, most articles are only published in one newspaper (*n* = 9522 ). The remaining articles are either published in two (n=1790 ), three (*n* = 2949 ), four (*n* = 2519) or in all five newspapers (*n* = 2905 ). A publication receives *M* = 287 (*SD* = 2038) views on average. The aggregate median shelf-life (i.e., the time at which 90% of all views it will ever receive have been received) is 34 hours (MAD=32). The 90th percentile is used because the time between publication and last view is very long, averaging 382 hours (*SD* = 514), or about 16 days.

### Viewing Trajectory

**Figure 2.**
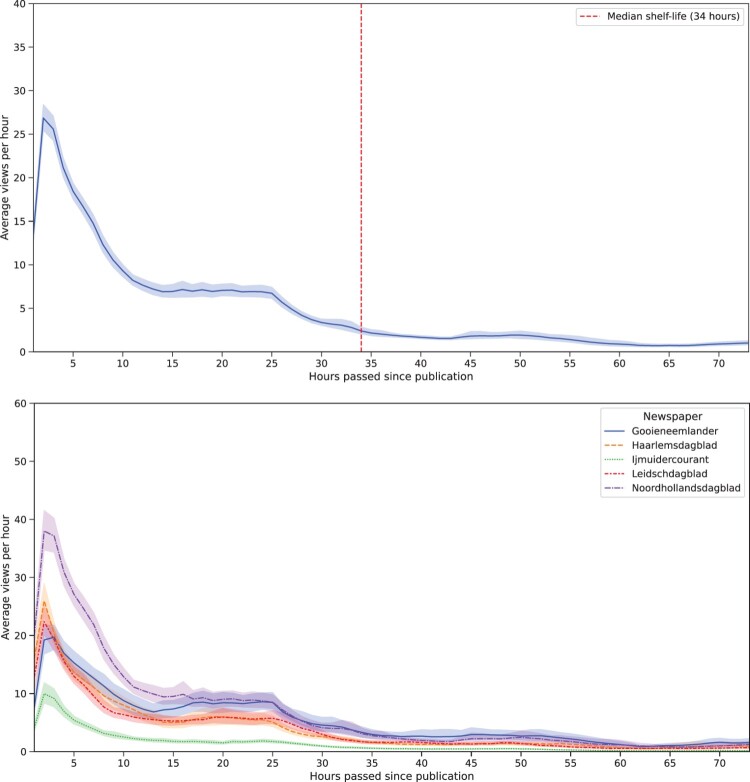
Viewing trajectory in the whole dataset (top) and per newspaper (bottom).

RQ1 asked how the number of people reading an article develops over time. Figure 2 shows that—on average—the maximum number of views is reached around two hours after publication, after which it declines quickly. Around the twelve-hour mark, it stabilizes until the 24-hour mark is reached. Thereafter, the number of views decreases to close to none.

The median shelf-life differs substantially between the newspapers with 14 hours being the shortest and 72 hours being the longest, with a median of 34 hours across all newspapers. However, the overall viewing trajectory appears to be very similar across the newspapers, as depicted in Figure 2.

### Relation between Character Count and Shelf-Life

H1 stated that there is a positive relationship between the length of an article and its shelf-life. The Spearman's rank correlation coefficient supports this hypothesis (*ρ*(46,548) =.17,p<.001) . The effect holds up in the multilevel model (Table 1): We estimate that all else being equal, an extra 1000 characters is associated with an extension of shelf life of between one and seven hours (*b* = .004, CI[.001;.007] ). This is in line with H1: Longer articles have a longer period in which they are read, while short articles are most-read immediately after publication.

**Table 1.** Mixed linear model predicting shelf life.

	Base model	Intermediate model	Theoretical model
Intercept	91.09(19.59)***	105.45(19.65)***	178.93(24.53)***
Newspaper (ref. = Gooi- en Eemlander)			
Haarlems Dagblad	53.19(5.16)***	49.25(5.19)***	48.47(5.16)***
Ijmuider Courant	−8.20(4.64)	−7.45(4.64)	−18.61(4.66)***
Leidsch Dagblad	43.43(5.16)***	37.42(5.22)***	30.94(5.21)***
Noordhollands Dagblad	225.33(4.44)***	191.64(6.76)***	182.73(6.80)***
Author (ref. = news agency)			
editor(s)	−41.78(18.73)*	−28.64(18.77)	−36.78(19.06)
individual reporter(s)	45.38(15.33)**	39.68(15.34)*	25.35(15.54)
our reporter(s)	53.30(19.82)**	51.58(19.82)**	31.72(19.94)
unknown	17.00(19.35)	14.59(19.35)	1.41(19.44)
Day of publication (ref. = Sunday)			
Monday	46.30(8.90)***	44.20(8.90)***	43.91(8.85)***
Tuesday	34.92(8.82)***	32.48(8.83)***	33.74(8.77)***
Wednesday	47.56(8.75)***	43.67(8.76)***	44.51(8.71)***
Thursday	40.02(8.77)***	35.98(8.79)***	37.02(8.74)***
Friday	46.16(8.62)***	41.08(8.64)***	38.62(8.58)***
Saturday	21.88(9.36)*	19.30(9.36)*	21.40(9.34)*
Time of publication (ref. = night)			
early morning	−14.02(9.66)	−27.75(9.86)**	−21.32(9.82)*
late morning	−5.78(8.92)	−21.87(9.26)*	−14.04(9.24)
early afternoon	−1.60(8.79)	−18.75(9.18)*	−14.46(9.13)
late afternoon	−8.31(8.64)	−25.59(9.07)**	−21.42(9.01)*
early evening	3.82(9.12)	−14.18(9.56)	−7.88(9.52)
late evening	−12.44(11.30)	−27.14(11.56)*	−20.86(11.49)
Inline_media	6.49(6.11)	8.92(6.11)	4.10(6.47)
Paid_content	−8.76(7.38)	−9.79(7.37)	−15.55(7.67)*
Section			
Culture	−1.95(22.45)	−4.11(22.45)	40.35(23.86)
Domestic	−45.64(16.80)**	−54.06(16.83)**	−25.43(18.80)
Foreign	−45.24(17.19)**	−53.15(17.21)**	−24.69(19.14)
Lifestyle	34.66(16.84)*	33.25(16.84)*	75.89(18.80)***
Opinion	−59.13(14.48)***	−57.77(14.48)***	44.00(17.18)*
Regional	−59.78(9.20)***	−50.72(9.25)***	28.32(12.27)*
Sport	−26.73(10.66)*	−29.66(10.66)**	26.27(12.34)*
Frontpage			−96.60(5.25)***
Recommended			125.07(34.71)***
Highlighted			−29.84(11.79)*
Background			97.02(17.89)***
Character count			0.00(0.00)**
Number of sections excl. frontpage			−99.73(17.12)***
Popularity rank in 1st 2h		0.27(0.04)***	0.18(0.04)***
Views in 1st 2h		−0.08(0.01)***	−0.04(0.01)***
*N*	37,028	37,028	37,028
$R_{\mathrm{marginal}}^{2}$	0.09	0.10	0.11
$R_{\mathrm{conditional}}^{2}$	0.28	0.29	0.30
*LL*	−264,425.85	−264,374.51	−264,156.92

*p < 0.05, **p < 0.01, ***p < 0.001.

Following our reasoning that character count is a proxy for in-depth rather than breaking news coverage, our findings are consistent with a similar pattern we find when examining the “Background” section. Moreover, articles that are published in the “Background” section appear to be significantly longer, having approximately twice as many characters (M=4989.49,SD=3893.92), compared to articles that are not published in this section (M=2465.79,SD=1965.60), *t*(46,548) =39.93,p<.001.

To validate, we manually coded *N* = 100 articles as background or non-background articles based only on title and teaser. To annotate the titles with teasers, we used the following question: *Does the news article focus primarily on the facts of a specific news event (0, non-background article) or does the news article attempt to place a news event or series of news events in a broader general or abstract context or trend (1, background article)?* Over a sample of 100 articles,^4^ we achieved a Krippendorff's *α* of.73 between our labeling and the website's. The articles we coded as background are indeed longer (M=5405.05,SD=3957.97) than others (*M* = 2484.52, *SD* = 1945.95), t(98)=4.33,p<.001.

### Relation between Number of Sections and Total Views

In line with H2, which predicted a positive relationship between the number of sections in which an article is published and its number of views, we find a positive relationship, *ρ*(46,548)=.62,p<.001 . Because publication in multiple sections will often include publication on the frontpage (r=.92), we constructed an additional variable that measures the number of sections excluding the frontpage. When we use this variable, we see a weaker but still significant positive relationship between the number of additional sections and views, *ρ*(46,548) =.15,p<.001 . This effect holds in the MLM model (B=424.43,p<.001, Table 2) in which the intercept is allowed to vary across article clusters, and in which we also include a dummy to control for publication on the homepage. This corroborates our finding that being published in more sections (beyond the frontpage) leads to more views overall, with the caveat that publication in more than two sections (beyond the frontpage) is relatively rare.

**Table 2.** Mixed linear model predicting total views.

	Base model	Intermediate model	Theoretical model
Intercept	181.08(76.23)*	−71.80(67.78)	−429.66(87.81)***
Newspaper (ref. = Gooi- en Eemlander)			
Haarlems Dagblad	−46.00(26.02)	−42.77(23.52)	−44.53(23.47)
Ijmuider Courant	−200.10(23.57)***	−125.38(21.25)***	−98.04(21.37)***
Leidsch Dagblad	−105.87(26.49)***	−71.01(24.09)**	−46.77(24.13)
Noordhollands Dagblad	−128.96(22.85)***	14.95(30.18)	30.39(30.36)
Author (ref. = news agency)			
editor(s)	1018.15(73.99)***	545.95(65.74)***	445.75(66.94)***
individual reporter(s)	56.28(59.52)	197.24(52.72)***	121.64(53.49)*
our reporter(s)	115.57(78.39)	68.58(69.47)	3.91(69.97)
unknown	60.50(75.24)	129.87(66.58)	64.95(66.92)
Day of publication (ref. = Sunday)			
Monday	−124.60(34.37)***	−85.89(30.41)**	−87.13(30.23)**
Tuesday	−112.95(34.18)**	−98.44(30.27)**	−95.32(30.07)**
Wednesday	−119.83(33.87)***	−64.10(30.02)*	−59.32(29.82)*
Thursday	−106.64(34.01)**	−59.87(30.16)*	−61.85(29.97)*
Friday	−140.28(33.37)***	−64.72(29.62)*	−58.92(29.42)*
Saturday	−81.75(36.12)*	−57.68(31.98)	−65.79(31.92)*
Time of publication (ref. = night)			
early morning	−15.16(36.99)	60.45(33.84)	39.35(33.71)
late morning	−1.59(33.97)	50.25(31.88)	28.03(31.85)
early afternoon	5.70(33.52)	64.47(31.75)*	44.97(31.57)
late afternoon	−10.03(32.85)	26.23(31.38)	15.21(31.19)
early evening	−58.55(34.78)	−34.28(33.19)	−50.42(33.02)
late evening	−9.87(42.96)	0.26(39.37)	−4.83(39.12)
Inline_media	99.19(24.40)***	12.70(21.65)	−8.28(22.87)
Paid_content	−90.20(29.25)**	−34.26(25.92)	−63.76(26.94)*
Section			
Culture	10.25(84.23)	31.80(74.36)	35.29(80.46)
Domestic	47.91(65.11)	221.48(57.70)***	128.48(65.93)
Foreign	39.75(66.49)	211.01(58.90)***	120.00(66.95)
Lifestyle	−1.78(63.12)	39.29(55.70)	23.58(63.92)
Opinion	242.44(54.30)***	208.93(47.92)***	15.57(59.55)
Regional	312.84(36.39)***	144.28(32.50)***	80.48(44.85)
Sport	51.65(41.91)	116.32(37.11)**	−34.63(44.81)
Frontpage			266.19(22.42)***
Recommended			300.20(122.17)*
Highlighted			−289.69(39.35)***
Background			−20.87(61.16)
Character count			0.00(0.01)
Number of sections excl. frontpage			424.43(66.11)***
Popularity rank in 1st 2h		−0.81(0.18)***	−0.52(0.18)**
Views in 1st 2h		4.05(0.04)***	3.97(0.04)***
*N*	37,028	37,028	37,028
$R_{\mathrm{marginal}}^{2}$	0.03	0.22	0.23
$R_{\mathrm{conditional}}^{2}$	0.06	0.24	0.25
*LL*	−322,478.62	−318,385.07	−318,234.18

*p < 0.05, **p < 0.01, ***p < 0.001.

### Relation Between Prominent Positions and Total Views

H3 stated that being presented as (a) published on the homepage, (b) as specifically highlighted, or (c) as recommended by the newsroom increases the number of views of an article. H3a is clearly supported (see also previous section): an article published on the frontpage gathers a much higher number of views (M=1027.99, SD=3916.64), in comparison to other articles (M=31.36, SD=189.37), t(46,548)=27.81 , p<.001. This effect holds in the multilevel model (B=266.19, p<.001); Table 2.

We regard two section types as constituting newsroom highlights. The first is named “Uitgelicht” (= Highlighted), and the second is named “Keuze van de redactie” (= Chosen by the editors). “Uitgelicht” can be accessed via the menu, but can also appear as specific boxes on the homepage as well as at other locations on the website. Similarly, “Keuze van de redactie” can be accessed directly via a dedicated URL (https://NEWSPAPER/keuze-van-de-redactie) but also via a box called “etalage” (= shop window) on the frontpage.

When a newspaper assigns one of these two tags to an article, it increases the article's potential exposure by: (1) making it accessible through multiple routes, and (2) signaling to readers that the story is particularly worthwhile via the word choice of the tags.

These different methods of highlighting an article yield different results. At first glance, being published on the *highlighted* page sustains more views (M=449.62, SD=1675.47), compared with not (M=369.12, SD=2308.62), t(46,548)=3.30, p=.001. However, when other variables are controlled for, this effect reverses and becomes significantly negative (B=−289.69, p<.001). Hence, we cannot unequivocally support H3b.

Being *chosen by the editors* (H3c), in contrast, has similar effect as being published on the homepage (H3a, see above). These specifically recommended articles gather more views than those who are not marked as such, t(46,548)=4.33, p<.001. This difference is substantial and holds up when controlled for in the multilevel model (B=300.20, p=.014).

We conclude that these techniques for promoting an article have a clear positive effect on the number of views–with the caveat that for articles that are promoted via the “highlighted” feature (as opposed to “chosen by the editors' or the frontpage), we obtain conflicting results depending on whether we include control variables or not.

### Views and Shelf-Life in Different Sections

**Figure 3.**
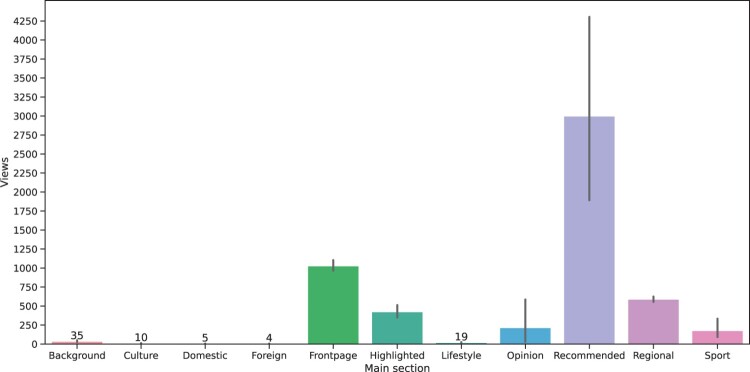
Average views per section. Note: The vertical lines in the middle of the bars show the 95% confidence interval for the mean values.

RQ2a asked how the popularity of different sections compares. Figure 3 illustrates that being recommended (by the editors) has the greatest impact on total views. Being published on the frontpage also results in a higher number of views compared with the other sections. The highlighted, opinion, regional and sports sections also gather a moderate amount of views. The remaining sections gather almost no views. These differences between sections are significant (h=34,852, p<.001).

**Figure 4.**
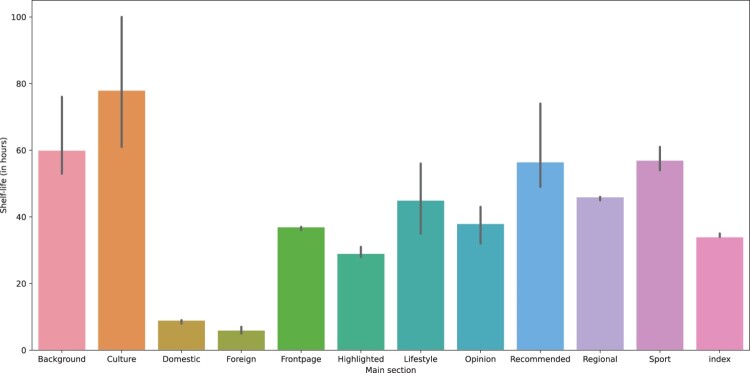
Median shelf-life per section. Note: The vertical lines in the middle of the bars show the 95% confidence interval for the median values.

RQ2b asked to what extent the section in which an article is published influences its shelf-life. We see that articles that are published in the Background and Culture section have a longer-lasting impact (see Figure 4). The frontpage and the highlighted section–two sections that suggest a prioritization of these articles–have a moderate lifespan. In particular domestic and foreign news has very short shelf-lives. Once again, these differences are significant (H=5879.63, p<.001).

We note that the “Recommended” section seems to be unique in increasing both shelf-life and total views. Others have very long shelf-life (Background, Culture) but a small number of views, or have, despite a high number of total views, only an average shelf-life (the frontpage).

### Influence of Popularity on Viewing Trajectory

RQ3 asked if initial popularity after publication could shape the overall viewing trajectory. Popularity in the first few hours can be operationalized either as absolute or as relative popularity.

Absolute popularity is measured by taking the sum of the views gathered within the first few hours. Given that the median value to reach the peak (the highest amount of views gathered in one hour during an article's lifespan) is 2 hours, we took the sum of views in the first two hours.

This absolute definition, however, does not consider that an article has to compete for views with other articles that are published within the same time frame. To account for this, we created a relative ranking. Within each one-hour time-slice, we ranked the articles based on the views gathered within that specific time frame. A value of 1 indicates that an article gathered the most views compared to other articles for that hour.

We illustrate the difference between absolute popularity (i.e., viewing amount) and relative popularity (i.e. popularity rank) with an example from our data. Within the first hour after publication, a specific article about an accident gathered 346 views. This is a very high amount: Only less than 1% of all articles did as well. Nonetheless, this article only has a popularity rank of eight: Seven articles gathered more views in the same time slot.

Absolute popularity (i.e., the total views within the first two hours) is positively correlated with shelf-life, ρ(46,548)=.14 , *p*<.001. Thus, the more views an article gathers in the beginning, the longer the article will stay relevant. However, in our multilevel model, the effect reverses and becomes significantly negative (B=−.05, p<.001).

The reason for this becomes clear when we take into account an article's competition in the first two hours. Doing so, the second result (more popularity early-on leads to a shorter shelf-life) is supported. There is a positive correlation between average popularity ranking in the beginning and shelf-life, ρ(46,548)=.31,p<.001 . In other words, the less popular an article is in the beginning (i.e., it is ranked, for example 30th instead of 1st), the higher its shelf-life will be. This effect holds in our multilevel model, B=.18, p<.001.

As both the bi-variate and the multivariate analysis of relative popularity, as well as the bi-variate analysis of absolute popularity point in the same direction, we take this as strong evidence that “peaking” early (in terms of views) tends to be associated with a decreased shelf-life of an article.

### Viewing Trajectory of Traffic Sources

RQ4 asked how external traffic influences the viewing trajectory of an article. As an initial exploration, the OLS mode in Table 3 shows the extent to which being distributed through each of the different traffic source (coded here as dummies; no views vs. views) contribute to total views. For instance, if an article is promoted through push messages, it can be expected to have 1641 additional views.^5^

**Table 3.** Effect of traffic source (no views vs. views) on total views.

	*B*	*SE*	*p*
Intercept	−145.2	41.9	0.001
Push	1641.8	36.0	<0.001
Newsletter	460.3	27.5	<0.001
Social	201.3	24.2	<0.001
Organic	92.6	20.8	<0.001
Other	69.5	40.3	0.084
Referral	−12.6	23.4	0.591

*F*(6, 46,543)=837.8, *p* < 0.001, $R^{2}=0.10$.

**Figure 5.**
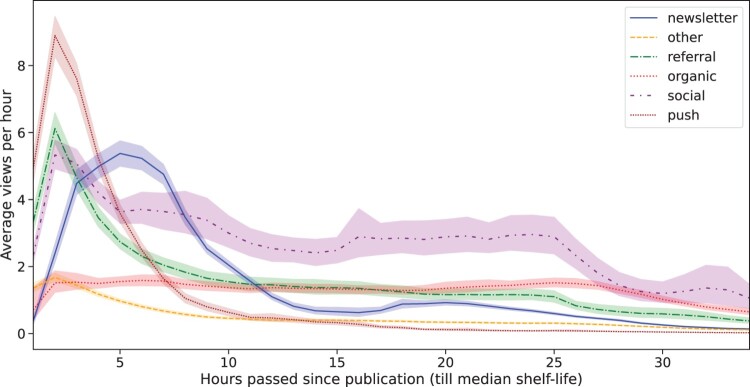
Traffic sources and Viewing trajectory (since publication).

But how does this develop over time? Figure 5 depicts the viewing trajectory of these different traffic sources. Push notifications seem to have an immediate effect on the viewing amount. However, this effect quickly wears off. The same is observed for the Web referrals, although the effect is smaller than that of push notifications.

Examining the URLs from which the referral clicks originate, three main categories can be determined: (i) social media (mainly Facebook, Twitter or LinkedIn), (ii) other local or national news websites, or (iii) one of the four news websites under study.

Views driven through the newsletter channel accumulate at a slower pace, taking both more time to peak and to decline. The only traffic source that has a *lasting* impact is social media. These are clicks that originate from promotional social media posts made by the news outlet online, and do not include clicks on articles shared by individual users (those fall under “referral”). Therefore, the effect we see here could underestimate the lasting impact of this promotional activity if it drives user activity such as re-shares.

**Figure 6.**
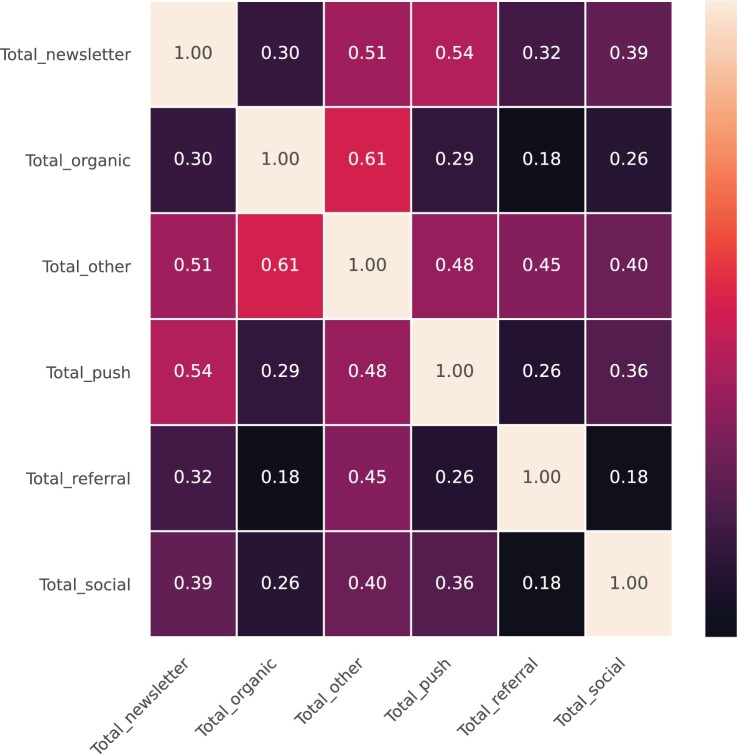
Correlations between standardized total views of traffic sources.

To determine whether different traffic sources reinforce or dampen each other, we test to what extent the total views gathered in each traffic source correlate with one another (Figure 6). Given that some traffic sources in general gather more views, the total views are standardized. By doing so, we test if the number of total views within each traffic source, relative to the average total views within each traffic source, co-vary. A high correlation means that if in a given time window for a given article, many views are attracted via one source, then this also happens via the other source. A low correlation means that how successful a source is has little to do with how successful the other one is.

The “other” category has relatively high correlations, but due to the ambiguous nature of “other”, we refrain from a substantive interpretation. Apart from that, the most substantially interesting correlation is between the standardized total views gathered through push and newsletter (*r* = .54). At the same time, this seems rather trivial given that a newspaper has the most control over sending out its news through push notifications and newsletters. Also an OLS model with interactions between traffic sources to predict total views shows significant yet very small interactions between most traffic sources, confirming our finding that reinforcment between channels is limited (see Supplementary Materials).

### The Role of Publication Time

While not a central focus of our study, we also examine the role of publication time as a control variable. The time of day that an article is published significantly predicts its shelf-life. Compared with articles published at night, those published in the early morning (B=−21.32, p<0.05), late afternoon (B=−21.42, p<0.05), or late evening (B=−20.86, p<0.10) exhibit a significantly shorter shelf life. Other times of day do not differ significantly from the nighttime baseline. On the other hand, the time of day of publication has no significant effect on the number of views it received.

## Conclusion and Discussion

We set out to study the viewing trajectories of individual news articles to shed light on the complex dynamics of online news popularity. From this examination, we can derive several insights.

First and foremost, there indeed are “winners” and “losers”: some articles draw an out-sized amount of attention, others almost none. While it may be intuitive to suspect that these differences are partly driven by bandwagon or “winner-takes-all” dynamics, our results paint a different picture.

It is not the case that articles that start out as popular compound this initial advantage (RQ3). On the contrary, their views decline sharper and they exhibit a shorter shelf-life. In absolute numbers, they may still receive more clicks than unpopular articles, also at later points in time—but they do not clearly profit from their early peak. Similarly, we find no evidence that unpopular articles may enjoy delayed attention that would allow them to “overtake” the first-movers. In sum, we do not see clear evidence of feedback-loop like processes in the popularity dynamics of individual articles over time.

Also interventions intended to kick off feedback-loop-like processes tend to have no lasting impact, with the notable exception of social media. A possible explanation could be that once links are lingering around on social media user's timelines and profiles, they stay around and still reach the extended networks of users after a time when the other activities' effects have evaporated. This has implications for newsrooms: Push messages, newsletters, and similar activities do have an effect and can be a good tool to keep users engaged (see Wheatley and Ferrer-Conill 2021)—but the boost is short-lived.

Our analysis underscores the complex nature of article popularity dynamics: The time of day when an article is published significantly influences its shelf life, with those published in the early morning, late afternoon, or late evening having a shorter lifespan compared to those published at night. However, publication time does not appear to significantly affect the total number of views an article receives. These results suggest that while publication time can impact the temporal distribution of views, it does not necessarily determine an article's overall popularity. This finding aligns with our others in sustaining that the popularity of online news articles is not driven by a simple, self-reinforcing feedback loop.

Broadly speaking, our results invite to re-consider what catering to audience demand means (see E. J. Lee and Tandoc 2017). As demand is not limited to short breaking-news articles, and as audiences seem to value editor's recommendations (see below), manual curation may in the long run be more important than popularity-based rankings, even though both may be combined in practice (Borchgrevink-Brækhus 2022). The feedback loop hypotheses would assume that popular articles attract more and more views over time—which does not seem to be the case, given that initial popularity does not appear to pay dividends in terms of sustained engagement. Partly, this may be because the news sites we studied only to a limited extent *additionally* amplify popular articles, such as via most-read boxes. This contrasts with A. M. Lee, Lewis, and Powers (2014) who found evidence for metric-driven feedback loops. However, in their observations, editors routinely and continuously adjust the ranking on the homepage by hand based on audience metrics—something that in the low-resource regional newspapers we study is much less likely to happen systematically (see Zamith 2016 who find conflicting results for a larger set of newspapers, including regional ones). If news sites, however, start using popularity to *automatically* re-rank articles on a large scale (see Borchgrevink-Brækhus 2022), this may change the picture. Future research needs to examine whether there is a point at which feedback loops kick in and override other factors, such as audience interests and journalistic choices.

We found very limited evidence that reinforcing processes act synergistically across different channels. With few exceptions, we could not confirm that, for instance, the increased views achieved through sending a push message would lead to higher views through other channels. The magnitude of the effect we observe seems to be too minor to be of practical importance.

Our preliminary explanation for the sharp decline in views associated with highly popular articles lies in the journalistic purpose that such articles often fulfill. Such articles may often regard “breaking news”, which attracts a lot of immediate interest but fades from relevance quickly. This explanation is supported by our finding that longer articles (which, in contrast to short braking news reports, encompass background, analysis, and feature stories) exhibit a flatter curve (H1): they may not attract as many views immediately but stay relevant longer. From a practical point of view, we suspect that breaking news essentially “exhausts” its audience (and relevance) quickly, whereas background articles take longer to reach saturation, but may present more sustained value to readers throughout this period. Consequently, both researchers and practitioners should distinguish between breaking news and background stories. As the latter seem to have the potential to attract readers for a longer time, it may be beneficial to prioritize them, even if they do not generate a large peak in clicks directly after publication.

Beyond different methods of attracting traffic to an article from those *outside* of the outlet's website (e.g., via push messages, newsletters, or social media) (RQ4), we find that the different placement of articles *within* the website also matters for the viewing trajectory of articles (RQ2). Homepage placement is a very strong driver of views (H3a). Vice versa, Bright and Nicholls (2014) suggest that journalists tend to keep often-clicked articles longer on the homepage. As we do not have data indicating the timestamp of removal from the homepage, we cannot test this hypothesis. Furthermore, being published on multiple places is not only common, but is also related to more views (H2). Moreover, news sites may influence the popularity of articles though editorial endorsement. However, the cues encoded in the mechanism of endorsement seem to matter. We observe that articles “chosen by the editor” are viewed more often, whereas those that are merely “highlighted” do not enjoy additional views beyond that which they would accumulate were they placed in any other additional section of the website beyond their initial placement.

### Reflections and Limitations

Studies like ours are necessary to better understand the changing agenda-setting role of journalists. While within the news sites we study, algorithmic ranking is limited to some “most read”-boxes, this is changing in many newsrooms. For instance, Borchgrevink-Brækhus (2022) describes in detail how on two Norwegian news sites only the first six articles are selected by human editors, while the other articles are ranked by an algorithm that is heavily driven by article popularity. This leaves less and less agency to journalists in actively shaping the agenda.

An important characteristic of the current study is that we had access to server-side log data. There is currently no other means of obtaining precise data on how often individual articles are clicked on within an actual news site. For example, other work uses data donations to understand use of online news (e.g., Loecherbach 2023; Wojcieszak et al. 2022). This can be a useful complement: It addresses the black spots our approach has on the user level and trades it for a black spot on the news site level, as the sample of data donation studies will never contain a large share of all users of a specific site.

While we studied regional newspapers in a small country, we believe that the general patterns we uncover should, at least to some extent, generalize to other news sites—not least because of the concentration on the media market, where (inter-)national conglomerates operate multiple of these sites, which therefore show large degree of overlap with other regional and national outlets. The Dutch media system is relatively similar to other (Northern-)European media systems. Traditional publishers still play a major role, also online. While our findings may not translate to other forms of online media (think of blogs or alternative media), there is little reason to expect that readers' behavior (and consequently, the shape of viewing trajectories over time) within a Dutch (regional) news website should be substantively different from readers' behavior on similar news websites in other countries. In contrast to this, we expect absolute numbers (like average number of views) to not generalize well (as also seen by the different coefficients for different newspapers). Cross-national research with countries in which news sites have different approaches to considering popularity (e.g., Norway: Borchgrevink-Brækhus 2022) may help further assessing generalizability.

Since the regional newspapers we studied are owned by the same parent company (as is often also the case in other markets), we were able to run sophisticated models in which we consider the inherent popularity of articles by focusing on identical stories that were published across multiple regional outlets. This greatly enhanced the robustness of our findings, compared to studying national outlets by different publishers, but could of course also be used in future work to model regional differences. We did not look systematically into these differences but noticed a tendency that smaller papers had flatter viewing trajectories, with (obviously) not only lower peaks, but interestingly, also longer shelf-lives. Hence, one may hypothesize that results generalize better to outlets of similar size; even though the general patterns still seem to be similar. Whilst beyond the scope of our current study, examining the exact content of the articles could lead to additional insights. Future work could conduct a content analysis of news articles to test, among other things, whether our proposed distinction between background and breaking news indeed holds up when examining the content in more detail; or how news values influence viewing trajectories (e.g., Boukes, Jones, and Vliegenthart 2022). Also, by analyzing the exact content, future work could go beyond our binary distinction between identical and different articles, and instead also consider a third category of articles that are *similar*, for instance adapted to a specific region.

## Supplementary Material

mh_appendix_rr2.pdf

## Data Availability

Due to legal and commercial restrictions, supporting data is not available.

## Notes

1 We use “views” and “clicks” interchangeably, because “viewing” an article typically implies “clicking” it first.

2 We ignore the edge case of zero pages where the article is still accessible if its URL is known.

3 We used the following categories; night (0≤h<6), early morning (6≤h<9), late morning (9≤h<12), early afternoon (12≤h<15), late afternoon (15≤h<18), early evening (18≤h<21) and late evening (21≤h<24).

4 Stratified sampling was used based on the background website label ($n_{\mathrm{non}-\mathrm{Background}}=43$, $n_{\mathrm{Background}}=57$) given the much higher prevalence of background over non-background articles.

5 This does not imply causality: Articles that are expected to only appeal to a niche audience are probably not selected to be promoted via push messages or newsletters.
