# Supplementary material for: What is Popular Gets More Popular? Exploring Over-Time Dynamics in Article Readership Using Real-World Log Data
Source: Journal Stud. 2024 Oct 14;25(16):2051–71. doi: 10.1080/1461670X.2024.2411334 (PMC11601048; doi:10.1080/1461670X.2024.2411334)
Supplement: mh_appendix_rr2.pdf [file RJOS_A_2411334_SM5384.pdf]

Appendix

Exploratory analyses

Additional features

Besides the predefined hypotheses and research questions, several other variables are relevant to explore. To begin with the time of the day. Naturally, little news is consumed in the early morning hours, after which you see an increase during breakfast. Also, a peak occurs during lunch and later in the day before dinner, after which views drop again in the later hours.

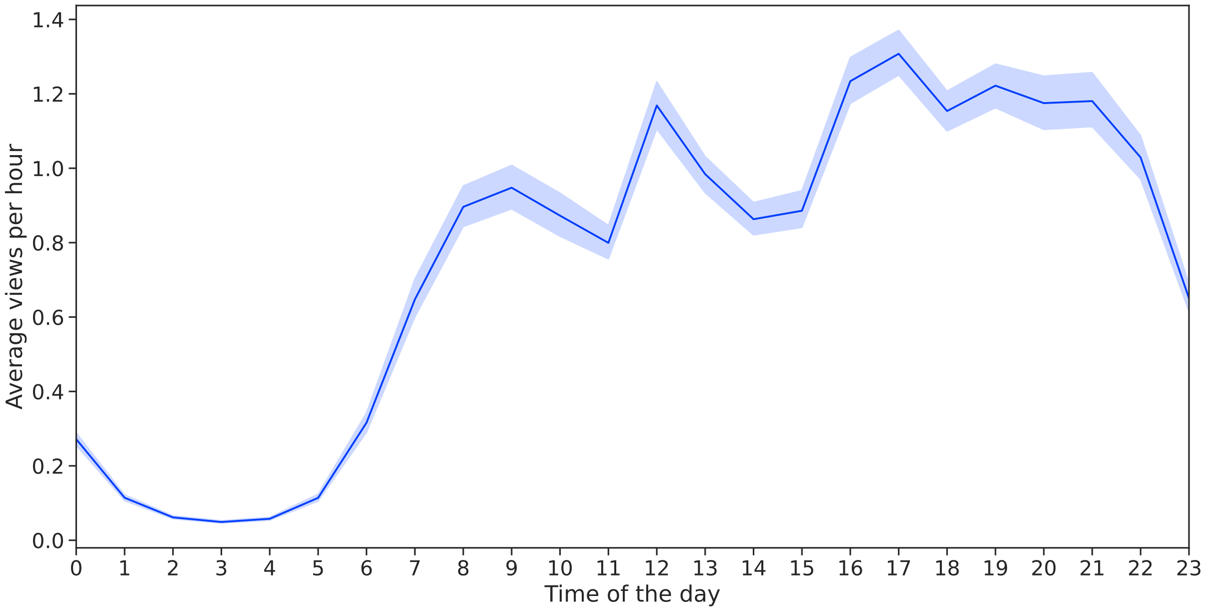

Figure 7
Views per Time of the Day

In the byline, there can be deducted who has written the article. The data indicates that this strongly influences the total views (see figure 8), $H = 22806.5$, $p < .001$. Especially articles that highlight that it is written by editors gather a high amount of views.

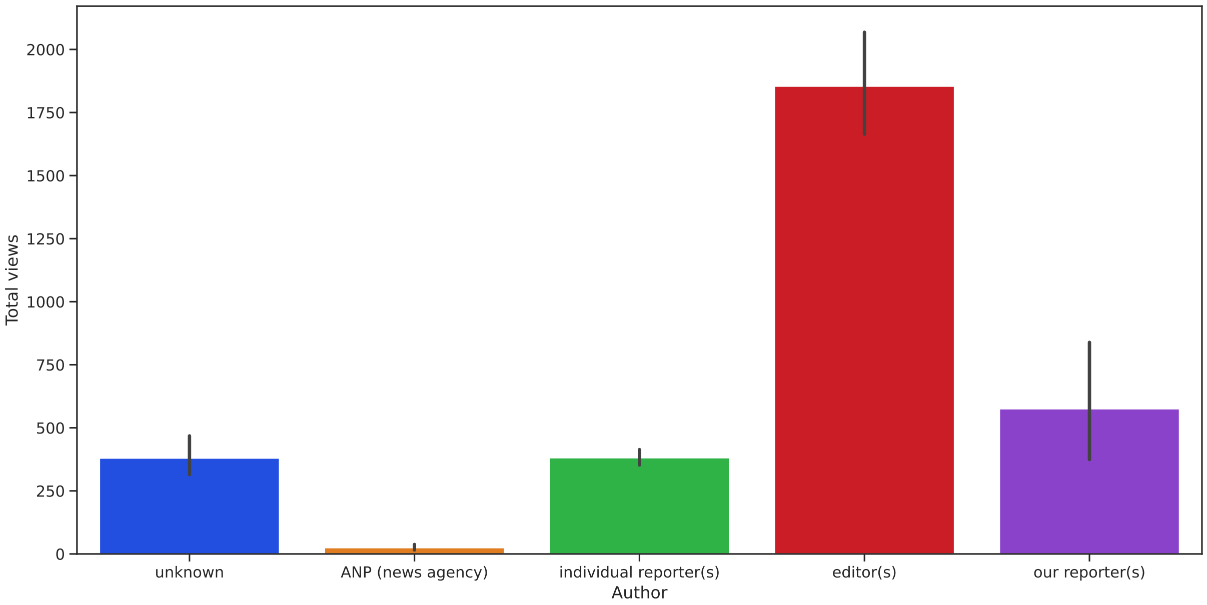

Figure 8
Byline and Total views

Note. The vertical lines in the middle of the bars show the 95% confidence interval for the mean values.

The third feature of interest is the presence of inline media. The results show that when an article contains inline media it receives more views (M = 688.13, SD = 2699.35), compared to when it does not (M = 173.82, SD = 1791.63), $t(46548)$ = -18.20, $p < .001$. Although this effect holds in the PRM, it does not in the OLS and MLM.[6]

Fourth is paid content. Paid content seems to last longer in terms of shelf-life (Med = 48.00) compared to free content (Med = 25.00), Z = 325486169.00, $p < .001$. This effect holds when controlled for.[7]

Lastly, an interesting pattern can be found when we look at the total views gathered per day (see figure9). On Monday a rather low amount of views is gathered, to then rise substantially on Tuesday. After that, the number of views consistently decreases during the workdays, with Friday having the lowest number of views across the week. At the weekend, however, the total views are substantially higher, with the Sunday being the absolute peak.

[6] OLS: B = 26.53, p = .246; PRM: B = .13, $p < .001$; MLM: B = -8.28, p = .718.

[7] OLS: B = -20.42, $p < .001$; MLM: B = -15.55, p = .043. Free content is in this the reference category.

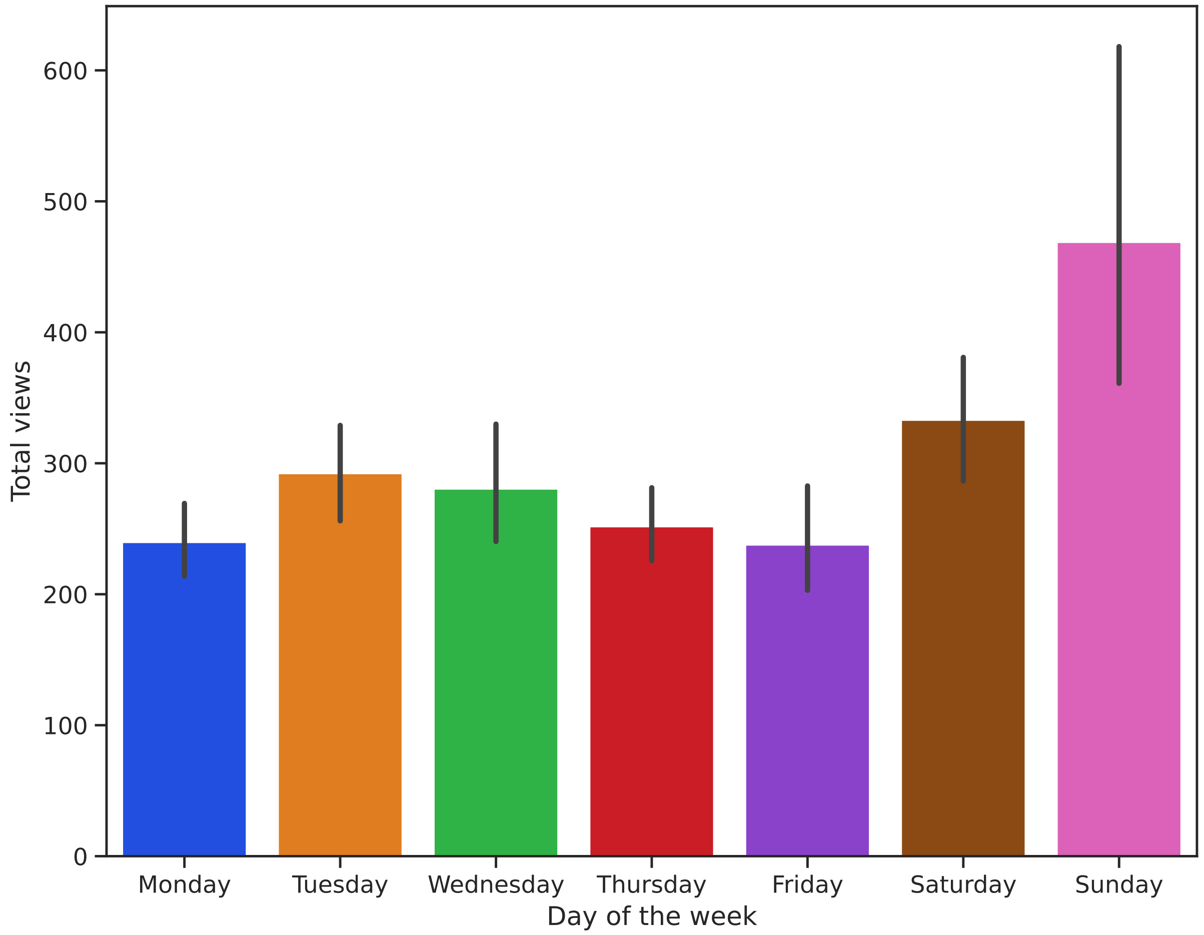

Figure 9

Day of the week and Total views

Note. The vertical lines in the middle of the bars show the 95% confidence interval for the mean values.

Predicting trajectory slopes

To get more understanding of what drives the shape of the viewing trajectory it can be deconstructed into two parts: the slope towards the peak (the hour where an article gathers the highest number of views), and the slope after the peak, till its shelf-life is reached. As we are at this point solely interested in the shape of the trajectory (is there a fast/slow increase/decrease) the slopes are based on the standardized views of each article.

We use these slopes as dependent variables in Ordinary least-squares Models to explore which features of an article impact its trajectory.

When looking at the highest coefficients, the slope towards the peak seems mostly to be affected by the topic of the article. Both background and culture for example, two rather in-depth topics, have a negative impact on the slope (background: B = -.69, p = .016; culture: B = -1.25, p = .002). This means that when an article is published on either the background or the culture page, it will take the article longer to reach its peak.

In addition, articles which are written by either individual reporters (B = -.86, $p <$ .001) or 'our reporters' (B = -1.14, $p <$ .001) (compared to news agencies) also take more time to reach their peak. Presumably, because articles written by reporters are also more in-depth compared to articles that originate from a news agency.

Being published on the front page, on the other hand, accelerates the slope towards the peak (B = 1.17, $p <$ .001).

Predicting the slope after the peak seems to be more problematic. The only noteworthy coefficient is that of the front page. Namely, whereas articles published on the front page peak sooner, they die off slower (B = -.26, $p <$ .001).

Regression models

Table 4

Ordinary Least Squares predicting shelf life

	Base model	Intermediate model	Theoretical model
**Intercept**	97.10 (14.61) ***	118.58 (14.59) ***	139.46 (16.86) ***
**Newspaper (ref. = Gooieneemlander)**			
**Haarlemsdagblad**	68.90 (5.55) ***	58.62 (5.56) ***	56.37 (5.53) ***
**Ijmuidercourant**	-8.06 (5.06)	-5.63 (5.05)	-18.22 (5.05) ***
**Leidschdagblad**	37.96 (5.20) ***	29.32 (5.21) ***	32.84 (5.18) ***
**Noordhollandsdagblad**	178.31 (4.60) ***	119.76 (5.84) ***	121.96 (5.88) ***
**Author (ref. = news agency)**			
**editor(s)**	-62.73 (13.04) ***	-36.80 (13.06) **	-28.27 (13.27) *
**individual reporter(s)**	36.32 (10.94) **	30.02 (10.89) **	21.77 (11.04) *
**our reporter(s)**	38.29 (14.02) **	36.45 (13.96) **	22.05 (14.05)
**unknown**	6.73 (13.90)	6.60 (13.83)	1.88 (13.90)
**Day of publication (ref. = Sunday)**			
**Monday**	49.28 (6.22) ***	45.36 (6.19) ***	43.22 (6.16) ***
**Tuesday**	44.54 (6.17) ***	38.26 (6.15) ***	39.44 (6.12) ***
**Wednesday**	57.68 (6.13) ***	49.33 (6.12) ***	50.10 (6.08) ***
**Thursday**	44.66 (6.16) ***	35.73 (6.15) ***	37.24 (6.11) ***
**Friday**	51.21 (6.06) ***	41.07 (6.06) ***	38.62 (6.02) ***
**Saturday**	29.44 (6.58) ***	23.75 (6.55) ***	24.62 (6.54) ***
**Time of publication (ref. = night)**			
**early morning**	-13.43 (7.10)	-40.02 (7.24) ***	-32.26 (7.21) ***
**late morning**	-2.38 (6.67)	-35.07 (6.93) ***	-24.53 (6.92) ***
**early afternoon**	-4.02 (6.57)	-38.22 (6.87) ***	-28.53 (6.85) ***
**late afternoon**	-10.20 (6.47)	-44.58 (6.80) ***	-36.10 (6.77) ***
**early evening**	4.55 (6.75)	-32.08 (7.10) ***	-20.54 (7.08) **
**late evening**	-20.45 (8.32) *	-49.73 (8.49) ***	-39.33 (8.45) ***
**Inline_media**	-0.48 (4.05)	2.94 (4.03)	-0.15 (4.30)
**Paid_content**	-16.47 (4.80) **	-16.87 (4.78) ***	-20.42 (4.98) ***
**Section**			
**Culture**	17.48 (16.77)	12.57 (16.70)	54.10 (17.72) **
**Domestic**	-43.40 (12.40) ***	-55.49 (12.36) ***	-19.31 (13.78)
**Foreign**	-42.72 (12.70) **	-54.00 (12.66) ***	-18.02 (14.05)
**Lifestyle**	50.09 (12.50) ***	46.41 (12.45) ***	85.68 (13.85) ***
**Opinion**	-47.48 (10.66) ***	-46.64 (10.61) ***	40.08 (12.11) **
**Regional**	-79.53 (6.87) ***	-59.08 (6.92) ***	17.69 (9.09)
**Sport**	-22.58 (7.54) **	-26.85 (7.51) ***	36.33 (8.73) ***
**Frontpage**			-89.02 (4.19) ***
**Recommended**			74.75 (21.26) ***
**Highlighted**			-10.14 (8.54)
**Background**			105.72 (13.16) ***
**Character_count**			0.00 (0.00) ***
**Number_of_sections_excl_frontpage**			-57.81 (8.04) ***
**Popularity_rank_in_1st_2h**		0.55 (0.03) ***	0.39 (0.03) ***
**Views_in_1st_2h**		-0.06 (0.01) ***	-0.04 (0.01) ***
N	46550	46550	46550
R^2	0.07	0.07	0.09
R^2_{adj}	0.07	0.07	0.08
F	112.82	120.05	117.59

Note. * $p < 0.05$, ** $p < 0.01$, *** $p < 0.001$.

Table 5
Poisson Model predicting shelf life

	Base model	Intermediate model	Theoretical model
**Intercept**	4.53 (0.00) ***	4.69 (0.00) ***	4.76 (0.00) ***
**Newspaper (ref. = Gooieneemlander)**			
**Haarlemsdagblad**	0.54 (0.00) ***	0.51 (0.00) ***	0.50 (0.00) ***
**Ijmuidercourant**	-0.12 (0.00) ***	-0.13 (0.00) ***	-0.19 (0.00) ***
**Leidschdagblad**	0.33 (0.00) ***	0.31 (0.00) ***	0.33 (0.00) ***
**Noordhollandsdagblad**	1.08 (0.00) ***	0.90 (0.00) ***	0.90 (0.00) ***
**Author (ref. = news agency)**			
**editor(s)**	-0.51 (0.00) ***	-0.24 (0.00) ***	-0.20 (0.00) ***
**individual reporter(s)**	0.20 (0.00) ***	0.14 (0.00) ***	0.11 (0.00) ***
**our reporter(s)**	0.22 (0.00) ***	0.17 (0.00) ***	0.13 (0.00) ***
**unknown**	0.04 (0.00) ***	0.02 (0.00) ***	0.02 (0.00) ***
**Day of publication (ref. = Sunday)**			
**Monday**	0.32 (0.00) ***	0.31 (0.00) ***	0.29 (0.00) ***
**Tuesday**	0.30 (0.00) ***	0.26 (0.00) ***	0.27 (0.00) ***
**Wednesday**	0.37 (0.00) ***	0.33 (0.00) ***	0.33 (0.00) ***
**Thursday**	0.30 (0.00) ***	0.26 (0.00) ***	0.26 (0.00) ***
**Friday**	0.34 (0.00) ***	0.29 (0.00) ***	0.27 (0.00) ***
**Saturday**	0.20 (0.00) ***	0.18 (0.00) ***	0.18 (0.00) ***
**Time of publication (ref. = night)**			
**early morning**	-0.08 (0.00) ***	-0.18 (0.00) ***	-0.14 (0.00) ***
**late morning**	-0.02 (0.00) ***	-0.13 (0.00) ***	-0.08 (0.00) ***
**early afternoon**	-0.03 (0.00) ***	-0.15 (0.00) ***	-0.10 (0.00) ***
**late afternoon**	-0.06 (0.00) ***	-0.18 (0.00) ***	-0.14 (0.00) ***
**early evening**	0.02 (0.00) ***	-0.11 (0.00) ***	-0.05 (0.00) ***
**late evening**	-0.14 (0.00) ***	-0.23 (0.00) ***	-0.18 (0.00) ***
**Inline_media**	-0.00 (0.00)	0.03 (0.00) ***	0.01 (0.00) ***
**Paid_content**	-0.08 (0.00) ***	-0.09 (0.00) ***	-0.10 (0.00) ***
**Section**			
**Culture**	0.05 (0.00) ***	0.02 (0.00) ***	0.32 (0.00) ***
**Domestic**	-0.26 (0.00) ***	-0.35 (0.00) ***	-0.06 (0.00) ***
**Foreign**	-0.26 (0.00) ***	-0.34 (0.00) ***	-0.05 (0.00) ***
**Lifestyle**	0.23 (0.00) ***	0.21 (0.00) ***	0.48 (0.00) ***
**Opinion**	-0.27 (0.00) ***	-0.26 (0.00) ***	0.26 (0.00) ***
**Regional**	-0.47 (0.00) ***	-0.35 (0.00) ***	0.07 (0.00) ***
**Sport**	-0.20 (0.00) ***	-0.20 (0.00) ***	0.24 (0.00) ***
**Frontpage**			-0.52 (0.00) ***
**Recommended**			0.52 (0.01) ***
**Highlighted**			-0.07 (0.00) ***
**Background**			0.54 (0.00) ***
**Character_count**			0.00 (0.00) ***
**Number_of_sections_excl_frontpage**			-0.37 (0.00) ***
**Popularity_rank_in_1st_2h**		0.00 (0.00) ***	0.00 (0.00) ***
**Views_in_1st_2h**		-0.00 (0.00) ***	-0.00 (0.00) ***
N	46550	46550	46550
AIC	15990247.11	15648487.37	15368786.62
BIC	15241240.68	14899498.43	14619850.18
LL	-7995093.55	-7824211.68	-7684355.31

Note. * $p < 0.05$, ** $p < 0.01$, *** $p < 0.001$.

Table 6

Ordinary Least Squares predicting total views

	Base model	Intermediate model	Theoretical model
**Intercept**	380.64 (92.96) ***	34.04 (77.96)	-338.21 (89.77) ***
**Newspaper (ref. = Gooieneemlander)**			
**Haarlemsdagblad**	-99.83 (35.30) **	-40.91 (29.71)	-38.96 (29.44)
**Ijmuidercourant**	-230.21 (32.19) ***	-142.93 (26.96) ***	-95.11 (26.88) ***
**Leidschdagblad**	-50.20 (33.08)	-26.96 (27.82)	-60.69 (27.58) *
**Noordhollandsdagblad**	51.66 (29.24)	274.11 (31.21) ***	234.75 (31.33) ***
**Author (ref. = news agency)**			
**editor(s)**	1148.90 (82.98) ***	187.93 (69.79) **	31.43 (70.67)
**individual reporter(s)**	-104.78 (69.57)	75.12 (58.20)	-52.13 (58.80)
**our reporter(s)**	60.71 (89.21)	-2.68 (74.61)	-93.18 (74.84)
**unknown**	-27.64 (88.40)	-36.58 (73.92)	-137.52 (74.04)
**Day of publication (ref. = Sunday)**			
**Monday**	-218.49 (39.56) ***	-127.56 (33.10) ***	-128.63 (32.81) ***
**Tuesday**	-214.45 (39.24) ***	-121.62 (32.86) ***	-122.96 (32.57) ***
**Wednesday**	-207.63 (38.99) ***	-77.27 (32.69) *	-73.95 (32.39) *
**Thursday**	-227.76 (39.16) ***	-94.86 (32.84) **	-105.63 (32.56) **
**Friday**	-237.17 (38.57) ***	-75.28 (32.37) *	-71.37 (32.08) *
**Saturday**	-114.09 (41.83) **	-57.42 (35.02)	-80.82 (34.80) *
**Time of publication (ref. = night)**			
**early morning**	-39.11 (45.15)	128.96 (38.68) **	82.39 (38.41) *
**late morning**	-52.95 (42.41)	96.78 (37.04) **	39.29 (36.85)
**early afternoon**	12.59 (41.82)	172.89 (36.70) ***	110.64 (36.45) **
**late afternoon**	-38.29 (41.16)	62.44 (36.32)	21.81 (36.03)
**early evening**	-96.10 (42.95) *	26.24 (37.95)	-32.88 (37.70)
**late evening**	-15.71 (52.89)	37.02 (45.36)	8.89 (45.00)
**Inline_media**	251.89 (25.73) ***	92.73 (21.55) ***	26.53 (22.88)
**Paid_content**	-111.05 (30.56) ***	-35.03 (25.56)	-110.57 (26.49) ***
**Section**			
**Culture**	-30.63 (106.71)	13.40 (89.25)	60.86 (94.35)
**Domestic**	-85.60 (78.90)	133.25 (66.06) *	4.04 (73.41)
**Foreign**	-102.43 (80.82)	114.40 (67.65)	-9.58 (74.82)
**Lifestyle**	-51.50 (79.54)	36.47 (66.52)	41.68 (73.74)
**Opinion**	285.65 (67.80) ***	234.78 (56.70) ***	31.43 (64.47)
**Regional**	450.25 (43.68) ***	160.92 (36.98) ***	78.51 (48.39)
**Sport**	-54.23 (47.99)	44.31 (40.15)	-165.15 (46.47) ***
**Frontpage**			375.22 (22.33) ***
**Recommended**			1586.13 (113.21) ***
**Highlighted**			-481.30 (45.51) ***
**Background**			-63.46 (70.09)
**Character_count**			0.01 (0.01) **
**Number_of_sections_excl_frontpage**			469.06 (42.81) ***
**Popularity_rank_in_1st_2h**		-2.31 (0.18) ***	-1.49 (0.18) ***
**Views_in_1st_2h**		4.42 (0.03) ***	4.32 (0.03) ***
N	46550	46550	46550
R^2	0.04	0.33	0.34
R^2_{adj}	0.04	0.33	0.34
F	68.71	737.19	653.95

Note. * $p < 0.05$, ** $p < 0.01$, *** $p < 0.001$.

Table 7

Poisson Model predicting total views

	Base model	Intermediate model	Theoretical model
**Intercept**	6.16 (0.00) ***	6.44 (0.00) ***	4.93 (0.00) ***
**Newspaper (ref. = Gooieneemlander)**			
**Haarlemsdagblad**	-0.49 (0.00) ***	-0.25 (0.00) ***	-0.13 (0.00) ***
**Ijmuidercourant**	-1.69 (0.00) ***	-1.58 (0.00) ***	-1.10 (0.00) ***
**Leidschdagblad**	-0.29 (0.00) ***	-0.20 (0.00) ***	-0.29 (0.00) ***
**Noordhollandsdagblad**	-0.08 (0.00) ***	1.14 (0.00) ***	1.07 (0.00) ***
**Author (ref. = news agency)**			
**editor(s)**	0.80 (0.00) ***	0.34 (0.00) ***	0.26 (0.00) ***
**individual reporter(s)**	-0.28 (0.00) ***	0.13 (0.00) ***	0.05 (0.00) ***
**our reporter(s)**	0.09 (0.00) ***	-0.09 (0.00) ***	0.01 (0.00) ***
**unknown**	-0.18 (0.00) ***	-0.13 (0.00) ***	-0.09 (0.00) ***
**Day of publication (ref. = Sunday)**			
**Monday**	-0.60 (0.00) ***	-0.44 (0.00) ***	-0.31 (0.00) ***
**Tuesday**	-0.53 (0.00) ***	-0.24 (0.00) ***	-0.20 (0.00) ***
**Wednesday**	-0.52 (0.00) ***	-0.18 (0.00) ***	-0.12 (0.00) ***
**Thursday**	-0.60 (0.00) ***	-0.19 (0.00) ***	-0.17 (0.00) ***
**Friday**	-0.66 (0.00) ***	-0.19 (0.00) ***	-0.16 (0.00) ***
**Saturday**	-0.24 (0.00) ***	-0.15 (0.00) ***	-0.14 (0.00) ***
**Time of publication (ref. = night)**	-0.95 (0.00) ***		-0.91 (0.00) ***
**early morning**	-0.95 (0.00) ***	-0.75 (0.00) ***	-0.91 (0.00) ***
**late morning**	-0.96 (0.00) ***	-0.59 (0.00) ***	-0.76 (0.00) ***
**early afternoon**	-0.76 (0.00) ***	-0.34 (0.00) ***	-0.60 (0.00) ***
**late afternoon**	-0.90 (0.00) ***	-0.59 (0.00) ***	-0.76 (0.00) ***
**early evening**	-1.10 (0.00) ***	-0.77 (0.00) ***	-0.96 (0.00) ***
**late evening**	-0.86 (0.00) ***	-0.70 (0.00) ***	-0.87 (0.00) ***
**Inline_media**	0.47 (0.00) ***	0.30 (0.00) ***	0.13 (0.00) ***
**Paid_content**	-0.25 (0.00) ***	-0.15 (0.00) ***	-0.43 (0.00) ***
**Section**			
**Culture**	-1.95 (0.02) ***	-1.73 (0.02) ***	-1.15 (0.02) ***
**Domestic**	-2.98 (0.00) ***	-2.72 (0.00) ***	-1.95 (0.01) ***
**Foreign**	-3.20 (0.01) ***	-2.99 (0.01) ***	-2.20 (0.01) ***
**Lifestyle**	-1.44 (0.01) ***	-1.16 (0.01) ***	-0.71 (0.01) ***
**Opinion**	1.31 (0.00) ***	1.28 (0.00) ***	0.83 (0.00) ***
**Regional**	1.93 (0.00) ***	1.23 (0.00) ***	0.87 (0.00) ***
**Sport**	-0.08 (0.00) ***	0.18 (0.00) ***	-0.27 (0.00) ***
**Frontpage**			1.97 (0.00) ***
**Recommended**			1.10 (0.00) ***
**Highlighted**			-0.73 (0.00) ***
**Background**			-0.78 (0.01) ***
**Character_count**			0.00 (0.00) ***
**Number_of_sections_excl_frontpage**			0.26 (0.00) ***
**Popularity_rank_in_1st_2h**		-0.03 (0.00) ***	-0.02 (0.00) ***
**Views_in_1st_2h**		0.00 (0.00) ***	0.00 (0.00) ***
N	46550	46550	46550
AIC	44985019.80	28688559.54	22540843.82
BIC	44279401.98	27982959.22	21835295.98
LL	-22492479.90	-14344247.77	-11270383.91

Note. * $p < 0.05$, ** $p < 0.01$, *** $p < 0.001$.

Table 8
Ordinary Least Squares predicting slope before peak

	Base model	Intermediate model	Theoretical model
**Intercept**	3.42 (0.31) ***	2.51 (0.31) ***	2.57 (0.34) ***
**Newspaper (ref. = Gooieneemlander)**			
**Haarlemsdagblad**	-0.25 (0.11) *	0.11 (0.10)	0.19 (0.10)
**Ijmuidercourant**	-0.79 (0.12) ***	-0.69 (0.12) ***	-0.52 (0.12) ***
**Leidschdagblad**	0.13 (0.10)	0.44 (0.10) ***	0.41 (0.10) ***
**Noordhollandsdagblad**	0.57 (0.09) ***	2.50 (0.12) ***	2.64 (0.12) ***
**Author (ref. = news agency)**			
**editor(s)**	1.15 (0.27) ***	0.37 (0.27)	0.51 (0.27)
**individual reporter(s)**	-1.24 (0.23) ***	-1.07 (0.23) ***	-0.86 (0.23) ***
**our reporter(s)**	-1.44 (0.29) ***	-1.43 (0.28) ***	-1.14 (0.29) ***
**unknown**	-0.80 (0.30) **	-0.89 (0.30) **	-0.70 (0.30) *
**Day of publication (ref. = Sunday)**			
**Monday**	0.60 (0.13) ***	0.74 (0.13) ***	0.77 (0.13) ***
**Tuesday**	0.39 (0.13) **	0.62 (0.13) ***	0.64 (0.13) ***
**Wednesday**	0.36 (0.13) **	0.67 (0.12) ***	0.70 (0.12) ***
**Thursday**	0.51 (0.13) ***	0.82 (0.12) ***	0.84 (0.12) ***
**Friday**	0.17 (0.13)	0.53 (0.12) ***	0.58 (0.12) ***
**Saturday**	0.14 (0.14)	0.34 (0.13) *	0.37 (0.13) **
**Time of publication (ref. = night)**			
**early morning**	-0.11 (0.15)	0.93 (0.16) ***	0.80 (0.15) ***
**late morning**	0.63 (0.14) ***	1.88 (0.15) ***	1.77 (0.15) ***
**early afternoon**	0.03 (0.14)	1.34 (0.15) ***	1.23 (0.15) ***
**late afternoon**	0.61 (0.14) ***	1.91 (0.15) ***	1.84 (0.15) ***
**early evening**	0.23 (0.15)	1.63 (0.15) ***	1.53 (0.15) ***
**late evening**	-0.22 (0.18)	0.97 (0.18) ***	0.84 (0.18) ***
**Inline_media**	0.45 (0.08) ***	0.35 (0.08) ***	0.25 (0.08) **
**Paid_content**	0.47 (0.10) ***	0.47 (0.09) ***	0.39 (0.10) ***
**Section**			
**Culture**	-1.54 (0.40) ***	-1.28 (0.40) **	-1.25 (0.41) **
**Domestic**	-1.26 (0.27) ***	-0.67 (0.26) *	-0.49 (0.28)
**Foreign**	-1.60 (0.27) ***	-1.07 (0.27) ***	-0.86 (0.29) **
**Lifestyle**	-0.86 (0.31) **	-0.65 (0.30) *	-0.77 (0.32) *
**Opinion**	-0.42 (0.24)	-0.43 (0.24)	-0.84 (0.26) **
**Regional**	1.67 (0.15) ***	0.98 (0.14) ***	0.43 (0.18) *
**Sport**	-0.69 (0.15) ***	-0.48 (0.15) **	-0.84 (0.17) ***
**Frontpage**			1.17 (0.08) ***
**Recommended**			-0.06 (0.38)
**Highlighted**			0.50 (0.17) **
**Background**			-0.69 (0.29) *
**Character_count**			0.00 (0.00)
**Number_of_sections_excl_frontpage**			-0.40 (0.15) **
**Popularity_rank_in_1st_2h**		-0.02 (0.00) ***	-0.02 (0.00) ***
**Views_in_1st_2h**		0.00 (0.00) ***	0.00 (0.00) ***
N	29936	29936	29936
R^2	0.08	0.11	0.12
R^2_{adj}	0.08	0.11	0.12
F	86.94	121.58	109.06

Note. * $p < 0.05$, ** $p < 0.01$, *** $p < 0.001$.

Table 9

Ordinary Least Squares predicting slope after peak

	Base model	Intermediate model	Theoretical model
**Intercept**	-0.36 (0.03) ***	-0.26 (0.03) ***	-0.15 (0.04) ***
**Newspaper (ref. = Gooieneemlander)**			
**Haarlemsdagblad**	-0.00 (0.01)	-0.03 (0.01) **	-0.05 (0.01) ***
**Ijmuidercourant**	0.05 (0.01) ***	0.05 (0.01) ***	-0.01 (0.01)
**Leidschdagblad**	0.02 (0.01)	0.00 (0.01)	0.01 (0.01)
**Noordhollandsdagblad**	0.06 (0.01) ***	-0.13 (0.01) ***	-0.17 (0.01) ***
**Author (ref. = news agency)**			
**editor(s)**	0.04 (0.03)	0.12 (0.03) ***	0.10 (0.03) ***
**individual reporter(s)**	0.20 (0.02) ***	0.17 (0.02) ***	0.13 (0.02) ***
**our reporter(s)**	0.17 (0.03) ***	0.17 (0.03) ***	0.11 (0.03) ***
**unknown**	0.17 (0.03) ***	0.17 (0.03) ***	0.13 (0.03) ***
**Day of publication (ref. = Sunday)**			
**Monday**	-0.01 (0.01)	-0.03 (0.01) *	-0.04 (0.01) **
**Tuesday**	0.02 (0.01)	-0.00 (0.01)	0.00 (0.01)
**Wednesday**	0.04 (0.01) **	0.01 (0.01)	0.01 (0.01)
**Thursday**	0.05 (0.01) ***	0.02 (0.01)	0.01 (0.01)
**Friday**	0.09 (0.01) ***	0.05 (0.01) ***	0.04 (0.01) **
**Saturday**	0.06 (0.01) ***	0.04 (0.01) **	0.03 (0.01) *
**Time of publication (ref. = night)**			
**early morning**	-0.05 (0.02) **	-0.17 (0.02) ***	-0.13 (0.02) ***
**late morning**	-0.05 (0.02) *	-0.18 (0.02) ***	-0.15 (0.02) ***
**early afternoon**	-0.03 (0.02)	-0.17 (0.02) ***	-0.14 (0.02) ***
**late afternoon**	0.00 (0.02)	-0.14 (0.02) ***	-0.11 (0.02) ***
**early evening**	0.02 (0.02)	-0.13 (0.02) ***	-0.09 (0.02) ***
**late evening**	-0.00 (0.02)	-0.13 (0.02) ***	-0.09 (0.02) ***
**Inline__media**	-0.02 (0.01) **	-0.01 (0.01)	0.01 (0.01)
**Paid__content**	-0.02 (0.01) *	-0.03 (0.01) **	-0.01 (0.01)
**Section**			
**Culture**	0.09 (0.04) *	0.06 (0.04)	0.04 (0.04)
**Domestic**	0.22 (0.03) ***	0.16 (0.03) ***	0.12 (0.03) ***
**Foreign**	0.24 (0.03) ***	0.18 (0.03) ***	0.14 (0.03) ***
**Lifestyle**	0.06 (0.03) *	0.04 (0.03)	0.04 (0.03)
**Opinion**	-0.06 (0.02) **	-0.06 (0.02) **	0.03 (0.02)
**Regional**	-0.22 (0.01) ***	-0.15 (0.01) ***	-0.07 (0.02) ***
**Sport**	-0.07 (0.02) ***	-0.09 (0.02) ***	-0.01 (0.02)
**Frontpage**			-0.26 (0.01) ***
**Recommended**			0.11 (0.04) **
**Highlighted**			-0.09 (0.02) ***
**Background**			0.06 (0.03) *
**Character__count**			-0.00 (0.00)
**Number__of__sections__excl__frontpage**			-0.01 (0.02)
**Popularity__rank__in__1st__2h**		0.00 (0.00) ***	0.00 (0.00) ***
**Views__in__1st__2h**		-0.00 (0.00) ***	-0.00 (0.00) ***
N	35337	35337	35337
R^2	0.06	0.09	0.11
R^2_{adj}	0.06	0.09	0.11
F	73.21	109.71	123.28

Note. * $p < 0.05$, ** $p < 0.01$, *** $p < 0.001$.

Table 10

Effect of total views per source and their interaction on overall total views

	b	SE
Intercept	-0.0057	0.001
newsletter	0.0871	0.002
organic	0.1946	0.002
newsletter:organic	-0.0044	0.000
other	0.0331	0.003
newsletter:other	-0.0079	0.000
push	0.1017	0.002
newsletter:push	0.0084	0.000
referral	0.3185	0.002
newsletter:referral	0.0065	0.000
social	0.5723	0.003
newsletter:social	0.0128	0.000
organic:other	0.0133	0.000
organic:push	0.0024	0.000
organic:referral	-0.0122	0.000
organic:social	-0.0029	0.000
other:push	-0.0073	0.000
other:referral	0.0130	0.000
other:social	-0.0032	0.000
push:referral	-0.0117	0.000
push:social	-0.0098	0.000
referral:social	-0.0057	0.000

Note. OLS regression to predict the total views gathered by the interactions between the standardized total views gathered within each traffic source. $F(21, 46528) = 35,070$, $p < .001$, $R^2 = 0.94$. All coefficients' p values $< .001$.
